# A meta-analytic study of partner phubbing and its antecedents and consequences

**DOI:** 10.3389/fpsyg.2025.1561159

**Published:** 2025-05-13

**Authors:** Nie Ni, Seyedali Ahrari, Zeinab Zaremohzzabieh, Mansoureh Zarean, Samsilah Roslan

**Affiliations:** ^1^Department of Educational Studies, Yuncheng University, Yuncheng, Shanxi, China; ^2^Preschool Education Research Center, Shanxi, China; ^3^Women and Family Studies Research Center, University of Religions and Denominations, Qom, Iran; ^4^Department of Social Science and Development Studies, Women Research Center, Alzahra University, Tehran, Iran; ^5^Faculty of Educational Studies, Universiti Putra Malaysia, Serdang, Malaysia

**Keywords:** antecedents and consequences, partner phubbing, romantic relationship, phubbing, phubbing behavior

## Abstract

Partner phubbing (Pphubbing)—the act of ignoring one’s romantic partner in favor of a smartphone or digital device—has become a widespread behavior, with detrimental effects on romantic relationships. This meta-analytic study synthesizes data from 52 studies (58 samples, *n* = 19,698) to examine both the antecedents and consequences of Pphubbing, providing a comprehensive understanding of its impact on relational dynamics and emotional well-being. The findings indicate that antecedents such as attachment anxiety, attachment avoidance, depression, and loneliness are significantly correlated with Pphubbing, with media addiction showing the strongest association (r_Z_ = 0.492). In contrast, self-esteem was not found to be a significant predictor of Pphubbing. Regarding the consequences, Pphubbing negatively affects several relational outcomes, including relationship satisfaction, marital satisfaction, romantic relationship quality, intimacy, responsiveness, and overall emotional closeness. It also contributes to increased conflict and heightened feelings of jealousy within relationships. These findings underscore the far-reaching implications of Pphubbing on both individual and relational well-being. We encourage future research to explore additional social, contextual, and psychological factors that may influence phubbing behaviors, and to investigate diverse types of digital interactions that may contribute to relational disruption in different cultural and situational contexts.

## 1 Introduction

In today’s digital age, smartphones have become indispensable tools in romantic relationships, enabling partners to stay connected, bridge long-distance gaps, and foster emotional closeness (Gomes et al., 2021). Research indicates that technology-mediated interactions, such as daily texting, can enhance relationship commitment, satisfaction, and communication (Aljasir, 2022). However, excessive smartphone use can also become a source of disruption, negatively impacting relationships by reducing quality time, increasing conflict, and leading to feelings of emotional neglect and decreased intimacy (Al-Saggaf, 2022; Arshad and Imran, 2022). This phenomenon, known as partner phubbing (Pphubbing), occurs when an individual prioritizes smartphone use over their partner during interactions, significantly diminishing relationship satisfaction (Beukeboom and Pollmann, 2021; Roberts and David, 2022).

Pphubbing has become a pervasive issue in modern relationships (Carnelley et al., 2023). Research by Roberts and David (2016) found that nearly half of their participants had experienced being phubbed by their romantic partner. This behavior acts as a social allergen, triggering increasingly negative reactions in the affected partner. Even the mere presence of a smartphone during face-to-face interactions can inhibit feelings of closeness and interpersonal trust, reducing empathy and understanding between partners (Roberts and David, 2016; Zhan et al., 2022). As a result, Pphubbing not only diminishes relationship quality but can also foster emotional disconnect, making it a serious concern in maintaining healthy romantic connections.

Several studies have examined both the antecedents and consequences of Pphubbing in relationships. Antecedents such as insecure attachment (Mosley, 2022), self-esteem (Wang et al., 2021), and jealousy (Arshad and Imran, 2022) have been linked to an increased likelihood of engaging in or perceiving Pphubbing behavior. The consequences of Pphubbing are typically associated with reduced relationship satisfaction, diminished relationship quality, and poorer emotional well-being (Chmielik and Błachnio, 2021), though most studies demonstrate correlational rather than causal relationships due to predominant cross-sectional designs.

However, research findings on Pphubbing show three specific inconsistencies: (1) variable effects (e.g., attachment anxiety showing stronger effects than avoidance in some studies but not others), (2) population differences (Western samples reporting more conflict while Eastern samples show greater marital satisfaction impacts), and (3) methodological variation (effect sizes ranging from 0.12 to 0.31 across study designs). These inconsistencies highlight the need for more comprehensive meta-analyses to clarify Pphubbing’s complex dynamics in romantic relationships. Given these gaps, a meta-analytic approach can statistically combine results to uncover patterns (Tamilmani et al., 2019). While prior meta-analyses examined Pphubbing in non-romantic contexts (e.g., parental phubbing; Lin et al., 2024) or smartphone addiction (Ansari et al., 2024), none analyzed romantic relationship outcomes specifically.

The current study aims to synthesize findings on the antecedents and consequences of Pphubbing in romantic relationships, with particular attention to potential cultural moderators. Specifically, it examines how cultural context influences (1) the strength of relationships between key predictors (e.g., attachment styles, media addiction) and Pphubbing, and (2) the magnitude of Pphubbing’s effects on relationship outcomes. By organizing these findings into an integrated framework, the study advances theoretical understanding while providing practical guidance for addressing Pphubbing across different cultural settings.

## 2 Literature review

Pphubbing, a combination of “partner” and “phubbing” (phone snubbing), describes the act of ignoring a romantic partner in favor of using a smartphone or digital device (Komnik, 2024; Lutz and Knop, 2020). This behavior has both behavioral and psychological dimensions, involving not just the physical act of engaging with a device but also the emotional consequences for the partner who feels neglected. Conceptually, Pphubbing disrupts face-to-face communication and can be perceived as a form of micro-betrayal, eroding trust and emotional intimacy. Over time, this can significantly diminish relationship quality and stability, as it is often associated with attachment issues, reduced relationship satisfaction, and increased dependence on technology.

To effectively measure Pphubbing, researchers have developed comprehensive scales that capture its frequency, context, and impact on relationships. One key tool is the Partner Phubbing Scale (PPS) created by Roberts and David (2016), which consists of nine items that assess how often individuals perceive being ignored by their partners due to device use, such as “*My partner glances at his/her cell phone when talking to me*.” Participants rate each item on a five-point Likert scale, and higher scores indicate higher levels of perceived Pphubbing. This scale has shown strong reliability and validity, with Cronbach’s α scores ranging from 0.80 to 0.89 across various studies and languages.

### 2.1 Antecedents of Pphubbing

In this study, a multi-theoretical framework was adopted, integrating Attachment Theory (Bowlby, 1975), Social Exchange Theory (Homans, 1958), a cognitive approach inspired by Cognitive Behavioral Theory (Beck, 1993), and Media Dependency Theory (MDT; Ball-Rokeach and DeFleur, 1976) to provide a comprehensive explanation of the antecedents of Pphubbing, while emphasizing the distinct role each theory plays in shaping this behavior. The framework focuses specifically on attachment anxiety and avoidance as key relational antecedents.

Attachment theory suggests that early life relationships shape adult attachment styles, which in turn affect how individuals engage in and respond to Pphubbing (Bröning and Wartberg, 2022). Those with high attachment anxiety tend to fear rejection and abandonment, making them particularly sensitive to perceived relationship threats such as Pphubbing (Bröning and Wartberg, 2022; Roberts and David, 2016). On the other hand, individuals with high attachment avoidance, who value independence and are uncomfortable with intimacy, may use Pphubbing as a way to create emotional distance or cope with neglect (Sun and Miller, 2023). Attachment theory thus highlights how emotional insecurity within relationships contributes to Pphubbing behavior, positioning it as a relational coping strategy.

SET complements this perspective by shifting the focus to the perceived costs and benefits of interpersonal relationships. SET posits that relationships are maintained through a cost–benefit analysis, where individuals seek to maximize rewards like emotional connection while minimizing costs such as neglect or rejection. In this context, low self-esteem, depression, and loneliness can be seen as psychological “costs” that make individuals more susceptible to the negative effects of Pphubbing (Chmielik and Błachnio, 2021). Individuals with low self-esteem may interpret Pphubbing as a sign of rejection, intensifying their emotional distress, while loneliness and depression exacerbate this relational strain as Pphubbing worsens feelings of isolation and detachment. These psychological factors were chosen due to their well-documented associations with emotional distress in relationships, making them highly relevant antecedents of Pphubbing.

The cognitive approach, drawing from Cognitive Behavioral Theory, emphasizes the role of maladaptive cognitions in shaping emotional and behavioral responses to Pphubbing. This perspective focuses on how negative thought patterns, such as “My partner’s phubbing means I’m unlovable,” amplify emotional distress. These cognitive distortions mediate the relationship between psychological vulnerabilities (e.g., low self-esteem, depression) and heightened sensitivity to Pphubbing, offering a micro-level complement to SET’s macro-level cost–benefit analysis. Unlike SET, which focuses on external relational dynamics, the cognitive approach highlights internal cognitive processes, such as negative automatic thoughts and dysfunctional beliefs about self-worth, rejection, or abandonment (Beck, 1993). For example, individuals prone to distorted cognitive patterns may perceive Pphubbing as intentional or threatening, reinforcing feelings of worthlessness or social exclusion. This cognitive lens provides a nuanced understanding of individual vulnerabilities to Pphubbing, distinguishing it from the interactional focus of SET.

Finally, MDT complements these psychological antecedents by explaining the compulsive nature of Pphubbing, as individuals become increasingly reliant on smartphones to fulfill personal needs for social connection, entertainment, or information (Ball-Rokeach and DeFleur, 1976). As smartphone dependency grows, individuals may engage in Pphubbing more frequently, leading to relational disruptions (Karaman and Arslan, 2024). This theory was chosen because it offers a comprehensive explanation of the underlying mechanisms driving problematic media use in relational contexts. Research supports a strong correlation between problematic media use and Pphubbing, with those heavily engaged in digital environments more likely to engage in Pphubbing (Harmon and Duffy, 2022).

### 2.2 Consequences of Pphubbing

In this study, Social Exchange Theory (SET), Attachment Theory, and Cognitive Dissonance Theory (CDT; Festinger, 1957) were employed to examine the relational and emotional consequences of Pphubbing in a theoretically integrated manner. Drawing from these frameworks, Pphubbing is shown to negatively impact individuals’ experiences of relational well-being (e.g., overall relationship quality, intimacy, and satisfaction) and emotional well-being (e.g., jealousy, insecurity, conflict, and diminished partner responsiveness) (Thomas et al., 2022).

Relational consequences can be best understood through the lens of SET, which emphasizes that individuals assess their relationships based on a cost–benefit analysis. When one partner engages in Pphubbing, the other often feels ignored or undervalued, leading to reduced emotional and relational “rewards” such as attention, responsiveness, and intimacy, and increased “costs” like neglect and frustration (David and Roberts, 2021). This imbalance adversely impacts relationship satisfaction, intimacy quality, and overall relational harmony. Rather than separating constructs like marital satisfaction, life satisfaction, and relationship satisfaction, this study conceptualizes them under the broader construct of relational satisfaction to reduce redundancy and better align with theoretical models (Roberts and David, 2016; Wang and Zhao, 2023; Yam, 2023). Thus, Pphubbing disrupts relational balance, decreasing the overall satisfaction derived from romantic interactions.

Emotional consequences, including jealousy, insecurity, and emotional disconnection, are effectively explained by Attachment Theory. Pphubbing threatens the sense of emotional security and trust within a relationship, particularly among individuals with anxious or avoidant attachment styles (Wang et al., 2024). The experience of being phubbed may elicit emotional rejection, activating attachment-related fears and increasing feelings of jealousy and vulnerability. Moreover, intimacy quality, a critical component of romantic relationships, is eroded as digital distractions interfere with meaningful connection and attentiveness. Frequent interruptions due to smartphone use diminish emotional closeness and perceived partner availability, further reducing relational satisfaction (Carnelley et al., 2023; Krasnova et al., 2016).

CDT offers additional insight into the interpersonal conflict and emotional dissonance associated with Pphubbing. According to CDT, individuals experience psychological discomfort when their expectations for emotional availability clash with the reality of digital disengagement. This discrepancy fosters frustration, unmet emotional needs, and interpersonal conflict (Roache, 2018; Zonash et al., 2020). Jealousy often arises when a partner perceives the smartphone as a competing attachment figure, while conflict results from repeated violations of emotional expectations. These emotionally charged interactions create a feedback loop of dissatisfaction, further escalating conflict and detachment.

Another significant consequence is the diminishment of partner responsiveness—the ability to recognize and empathetically respond to a partner’s emotional needs (Itzchakov et al., 2022). Pphubbing diverts attention away from dyadic interactions, impairing the partner’s ability to express support, validation, and empathy. The lack of responsiveness leads to emotional distance, diminished trust, and increased feelings of neglect and resentment. Consequently, emotional intimacy and relationship quality deteriorate.

**Figure 1:**
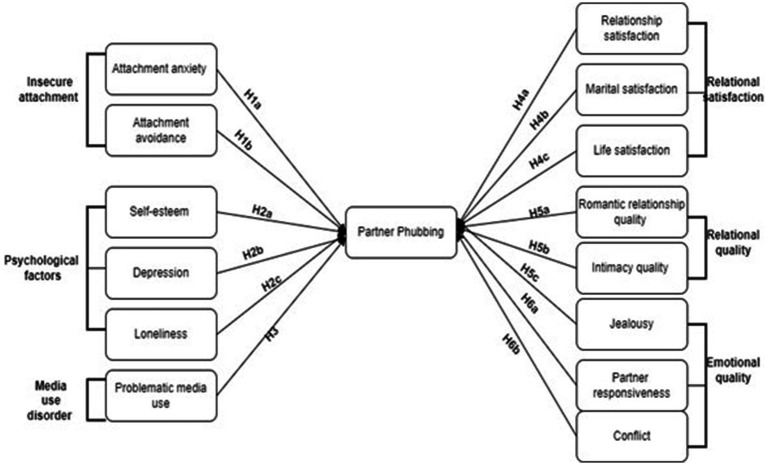
Hypothesized relationships. The model illustrates key predictors (attachment styles, psychological factors, media addiction) and consequences (relational, emotional, and satisfaction outcomes) of Pphubbing, along with hypothesized directional relationships (H_1_–H_6_).

Finally, research indicates that Pphubbing also intensifies conflict related to technology use, creating a cycle of retaliation and emotional withdrawal. As smartphone-related disagreements emerge, partners may resort to further Pphubbing—either passively as avoidance or actively as retaliation—which exacerbates emotional insecurity and contributes to relational dissatisfaction (David and Roberts, 2021). Based on this literature, a research model has been developed to explore the antecedents and consequences of Pphubbing, as illustrated in Figure 1.

### 2.3 Moderators

Numerous studies have investigated the relationship between Pphubbing and its effects, yet the findings are often inconsistent. These variations may stem from the presence of several moderating factors, such as age, gender, and cultural background, that influence the relationship between Pphubbing and its outcomes. Age serves as a significant moderator, with younger couples, particularly those in the early stages of their relationships, placing greater emphasis on constant communication, thereby being more susceptible to the negative effects of Pphubbing (Chmielik and Błachnio, 2021; Khodabakhsh and Le Ong, 2021). In contrast, older couples, who prioritize quality time and face-to-face interactions, may experience different emotional and relational consequences when Pphubbing occurs.

Gender is another moderating factor, as research shows that women are generally more prone to using social media and engaging in Pphubbing behaviors compared to men (Ivanova et al., 2020; Xie et al., 2022). Women also tend to use mobile phones to alleviate anxiety and maintain social connections, which may exacerbate the impact of Pphubbing on relationship satisfaction. Men, on the other hand, tend to display higher psychological resilience and are less affected by Pphubbing behaviors, reducing their dependence on mobile devices for emotional support.

The length of the relationship further moderates the effects of Pphubbing. Interdependence theory suggests that individuals’ outcomes are influenced by their partners’ behaviors, meaning both positive and negative experiences may be shared in long-term relationships (Rodriguez et al., 2014; Totenhagen et al., 2016). Consequently, in longer-term relationships, one partner’s behaviors, such as Pphubbing, are more likely to affect the other partner’s emotional and relational outcomes (Al-Saggaf and O’Donnell, 2019). This effect may be smaller or even non-existent in shorter-term relationships, making those in longer relationships more vulnerable to relationship dissatisfaction and depression in the face of Pphubbing (Wang et al., 2017).

Cultural background also moderates the relationship between Pphubbing and its effects. In collectivist societies, where interpersonal relationships and social harmony are highly valued, Pphubbing may be more disruptive due to the greater importance placed on maintaining close connections and in-person interactions (Błachnio et al., 2021). The widespread use of mobile devices, particularly among younger generations who have integrated these technologies into their daily lives, has further intensified the prevalence of Pphubbing. Identifying these moderating factors—age, gender, relationship length, and cultural context—provides a deeper understanding of Pphubbing’s varying impacts across different demographic and cultural groups.

## 3 Research methodology

To test our hypotheses, we conducted a meta-analysis in accordance with PRISMA guidelines and recommendations from prior research (Aytug et al., 2012; Hunter and Schmidt, 2014).

### 3.1 Identifying sources

The authors used multiple strategies to identify both published and unpublished studies that empirically examined parent phubbing. In August 2024, searches were conducted across databases including Google Scholar, Scopus, EBSCO, DOJA, PsycINFO, ScienceDirect, SpringerLink, JSTOR, Emerald, and ProQuest. The search strategy followed Boolean techniques, pairing keywords such as “partner phubbing” AND “attachment anxiety” OR “avoidance” OR “low self-esteem” OR “depression” OR “loneliness” OR “media addiction” OR “relational satisfaction” OR “marital satisfaction” OR “romantic relationship quality” OR “jealousy” OR “partner responsiveness.” We also contacted authors for unpublished data and conducted an unstructured Google search (Cooper et al., 2019). Finally, reference lists of relevant studies were reviewed to identify additional sources.

### 3.2 Inclusion criteria

**Figure 2:**
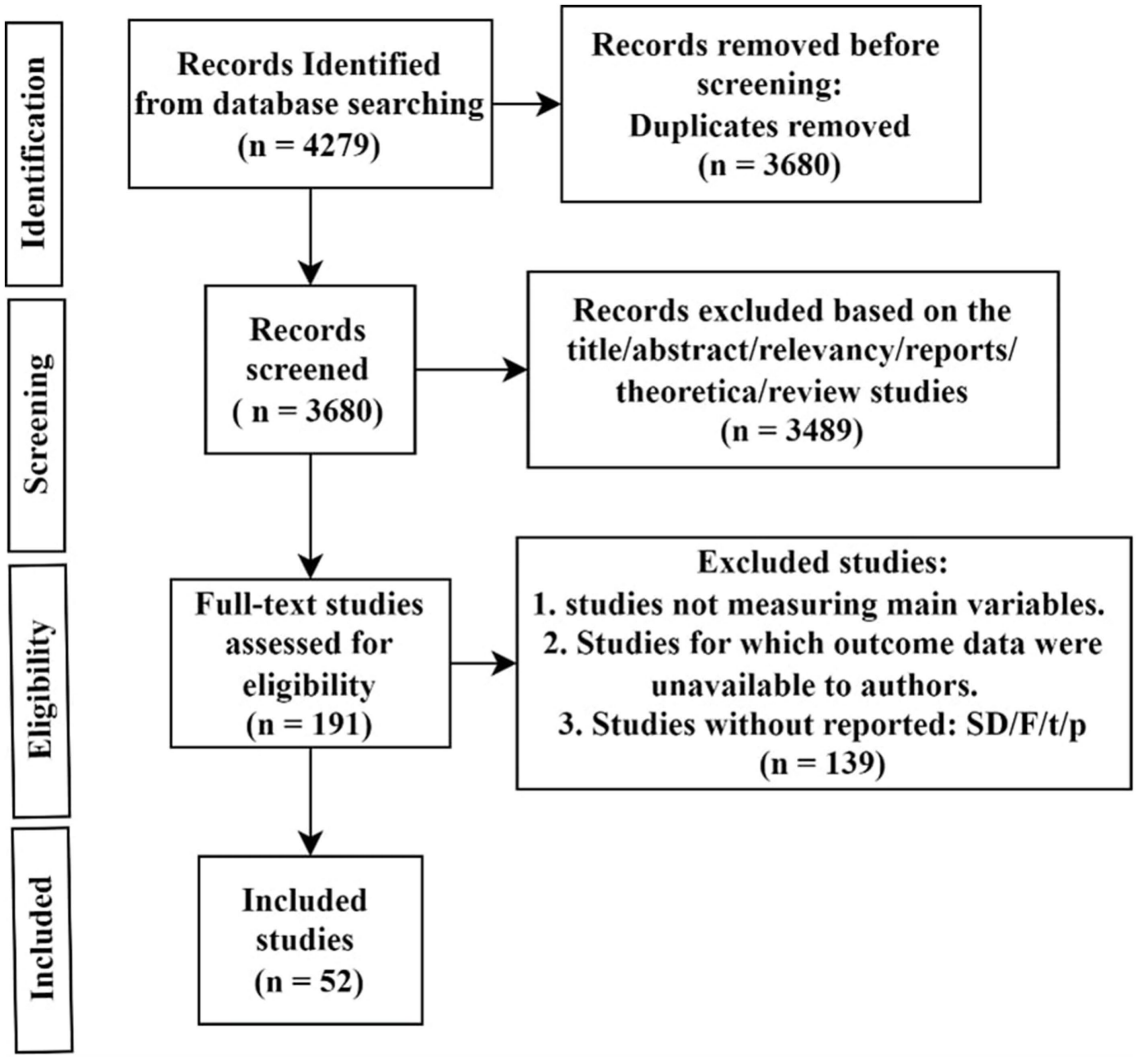
Flow chart for selection of included studies. The PRISMA-style flowchart depicts the study selection process, from initial database searches (*n* = 4,279) to final inclusion (*n* = 52), with exclusion criteria clearly specified.

A total of 4,279 sources, including articles, dissertations, theses, and conference proceedings, were initially identified. Each source was reviewed to ensure it met the following criteria: a sample was collected, Pphubbing was measured, and quantitative statistics were reported. After this screening, 191 sources remained. Hundred-thirty-nine sources were excluded due to not measuring the main variables, lack of available outcome data, or unreported statistical results. Two trained researchers independently coded and recorded effect sizes for the desired relationships. Ultimately, 52 datasets met the inclusion criteria for the meta-analysis (Figure 2).

### 3.3 Data analysis

In this meta-analysis, direct effects refer to the bivariate relationships between Pphubbing and its identified antecedents and consequences—such as attachment anxiety, attachment avoidance, self-esteem, loneliness, depression, relationship satisfaction, life satisfaction, marital satisfaction, jealousy, intimacy quality, conflict, and partner responsiveness. Each of these relationships was treated as a separate direct effect and analyzed independently.

The current authors conducted a separate analysis for each direct effect using Comprehensive Meta-Analysis (CMA) software. Effect sizes, typically correlation coefficients, were weighted by sample size or variance, and a random effects model was employed to calculate meta-analytic estimates, requiring at least three studies per relationship. This threshold was selected to ensure statistical reliability and avoid unstable effect size estimates based on too few data points, which can distort meta-analytic results (Valentine et al., 2010; Steel and Kammeyer-Mueller, 2002). Heterogeneity among studies was assessed using Q and *I*^2^ statistics. Significant Q-values and high *I*^2^ indicated heterogeneity. To examine potential sources of heterogeneity, moderator analyses were conducted using both subgroup comparisons and univariate meta-regression. Subgroup analyses focused on cultural context (Eastern vs. Western cultures) to explore how cultural norms influence the strength of relationships between Pphubbing and its antecedents or consequences. In addition, we used weighted least squares (WLS) meta-regression to examine the influence of continuous moderators, including relationship length (in years), percentage of female participants, and mean participant age, on key variables such as attachment dimensions, relationship satisfaction, marital satisfaction, intimacy, jealousy, and conflict. To address publication bias, we performed Rosenthal’s (1979) fail-safe N and Egger’s test.

### 3.4 Quality assessment

Two researchers independently evaluated the quality and validity of the 52 studies meeting the inclusion criteria, using the ‘Quality Assessment and Validity Tool for Correlational Studies’ (Cicolini et al., 2014). This tool assessed four key aspects: study design, sample selection, instruments used, and data analysis. Thirteen standards were considered, with a maximum score of 14 points. Studies were classified as low (0–4), moderate (5–9), or high (10–14) quality. Discrepancies in scoring were resolved through discussion. All 52 studies were ultimately rated as high-quality (Tables 1, 2).

**Table 1:** Summary of quality assessment.

No.	Study	FS	PS	SSS	SML	PP	SRR	RRM	RVM	VMI	DIC	TD	CAM	OM	Total
1	Ajooba and Ambarwati (2023)	1	0	1	0	1	1	1	1	1	2	0	1	1	11
2	Arshad and Imran (2022)	1	1	1	1	1	1	1	1	1	2	1	1	1	14
3	Aljasir (2022)	1	1	1	1	1	1	1	1	1	2	1	1	1	14
4	Abeele et al. (2019)	1	1	1	0	1	1	1	1	1	2	1	1	1	13
5	Allred (2020)	1	1	1	1	1	1	1	1	1	2	1	1	1	14
6	Beukeboom and Pollmann (2021)	1	1	1	1	1	1	1	1	1	0	1	1	1	12
7	Bröning and Wartberg (2022)	1	1	1	1	1	1	1	1	1	0	1	1	1	12
8	Booth et al. (2021)	1	1	1	1	1	1	1	1	1	2	1	1	0	13
9	Chmielik and Błachnio (2021)	1	1	1	0	1	1	1	1	1	2	0	1	0	11
10	Chotpitayasunondh and Douglas (2018)	1	1	1	0	1	1	1	1	1	2	1	1	0	12
11	Carnelley et al. (2023)	1	1	1	0	1	1	1	1	1	0	1	1	0	10
12	Çizmeci (2017)	1	1	1	0	1	1	0	1	1	2	1	1	0	11
13	David and Roberts (2021)	1	1	1	1	1	1	1	1	1	2	1	1	1	12
14	Etesami et al. (2023)	1	1	1	1	1	1	1	1	1	2	1	1	1	14
15	Farooqi et al. (2021)	1	1	1	1	1	1	1	1	1	2	1	1	1	14
16	Frackowiak et al. (2022)	1	1	1	1	1	1	1	1	1	2	1	1	1	14
17	Gomes et al. (2021)	1	1	1	1	1	1	1	1	1	2	1	1	1	14
18	Ivanova et al. (2020)	1	1	1	1	1	1	1	1	1	2	1	1	1	14
19	Ippolito (2020)	1	1	1	1	1	1	1	1	1	2	1	1	1	14
20	Johnson (2020)	1	1	1	1	1	1	1	1	1	2	1	1	1	14
21	Kadylak (2020)	1	1	1	1	1	1	1	1	1	2	1	1	1	14
22	Kılıçarslan and Parmaksız (2023)	1	1	1	1	1	1	1	1	1	2	1	1	1	14
23	Karaman and Arslan (2024)	1	1	1	1	1	1	1	1	1	2	1	1	1	14
24	Krasnova et al. (2016)	1	1	1	1	1	1	1	1	1	2	1	1	1	14
25	Khodabakhsh and Le Ong (2021)	1	1	1	1	1	1	1	1	1	2	1	1	1	14
26	Lapierre and Zhao (2024)	1	1	1	1	1	1	1	1	1	2	1	1	1	14
27	Lutz and Knop (2020)	1	1	1	1	1	1	1	1	1	2	1	1	1	14
28	Maftei and Măirean (2023)	1	1	1	1	1	1	1	1	1	2	1	1	1	14
29	Mahmud et al. (2024)	1	1	1	1	1	1	1	1	1	2	1	1	1	14
30	McDaniel and Drouin (2019)	1	1	1	1	1	1	1	1	1	1	1	1	1	13
31	McDaniel et al. (2018)	1	1	1	1	1	1	1	1	1	1	1	1	1	13
32	Mosley (2022)	1	1	1	1	1	1	1	1	1	1	1	1	1	13
33	Mosley and Parker (2023)	1	1	1	1	1	1	1	1	1	2	1	1	0	13
34	Parmaksız (2021)	1	1	1	1	1	1	1	1	1	2	1	1	0	13
35	Peleg and Boniel-Nissim (2024)	1	1	1	1	1	1	1	1	1	2	1	1	0	13
36	Rafiq (2023)	1	1	1	1	1	1	1	1	1	2	1	1	0	13
37	Roberts and David (2022)	1	1	1	1	1	1	1	1	1	2	1	1	0	13
38	Roberts and David (2016)	1	1	1	1	1	1	1	1	1	2	1	1	0	13
39	Schokkenbroek et al. (2022)	1	1	1	1	1	1	1	1	1	2	1	1	0	13
40	Sun and Samp (2022)	1	1	1	1	1	1	1	1	1	2	1	1	0	13
41	Thomas et al. (2022)	1	1	1	1	1	1	1	1	1	2	1	1	0	13
42	Togar et al. (2023)	1	1	1	1	1	1	1	1	1	2	1	1	0	13
43	Wang et al. (2024)	1	1	1	1	1	1	1	1	1	2	1	1	0	13
44	Wang et al. (2024)	1	1	1	1	1	1	1	1	1	2	1	1	0	13
45	Wang et al. (2017)	1	1	1	1	1	1	1	1	1	2	1	1	0	13
46	Wang and Zhao (2023)	1	1	1	1	1	1	1	1	1	2	1	1	0	13
47	Wang et al. (2021)	1	1	1	1	1	1	1	1	1	2	1	1	0	13
48	Xie et al. (2022)	1	1	1	1	1	1	1	1	1	2	1	1	0	13
49	Yam (2023)	1	1	1	1	1	1	1	1	1	2	1	1	0	13
50	Zhan et al. (2022)	1	1	1	1	1	1	1	1	1	2	1	1	0	13
51	Zhang et al. (2023)	1	1	1	1	1	1	1	1	1	2	1	1	0	13
52	Zonash et al. (2020)	1	1	1	1	1	1	1	1	1	2	1	1	0	13

FS, Future studies; PS, Probability sampling; SSS, Suitable sample size; SML, Sample from more than one location; PP, Privacy preserved; SRR, Suitable response rate; RRM, Result(s)’ reliable measurement; RVM, Result(s)’ valid measure; VMI, Valid measure of independent variables; DIC, Dependent variable internal consistency; TD, Theory driven; CAM, Correlation analysis for multiple effects; OM, Outliers’ management.

**Table 2:** An overview of the studies used in the meta-analysis processes.

Authors	Year	PT	Location	N	MA	MRL	RTP	G(F)%	Authors	Year	PT	Location	N	MA	MRL	RTP	G(F)%
Beukeboom and Pollmann ^1^	(2021)	JA	Netherlands	507	31.9	9	-	78.3	Roberts and David	(2016)	JA	USA	308	-	-	-	46
									Roberts and David	(2016)	JA	USA	145	-	-	-	55
Beukeboom and Pollmann ^2^	(2021)	JA	Netherlands	386	27.9	7	-	71.20	Rafiq	(2023)	TE	USA	193	-	-	-	-
Frackowiak et al.	(2022)	JA	Switzerland	133	33.7	10.3	-	51.9	Carnelley et al.	(2023)	JA	UK	100	-	2.84	14.5% Married	
Thomas et al.	(2022)	JA	UK	75	32	8	-	69.3	Mosley	(2022)	TE	USA	232	25	-	-	47
Mosley and Parker	(2023)	JA	USA	103	-	-	-	-	Peleg and Boniel-Nissim	(2024)	JA	Israel	431	29.05	6.06	-	66.6
Zonash et al.	(2020)	JA	Pakistan	120	-	-	Married	50	Yam	(2023)	JA	Türkiye	308	30.1	19 (6.2%)	-	78.90
Krasnova et al.	(2016)	CP	Germany	286	-	-	-	64	Wang et al.	(2017)	JA	China	243	-		Married	64.19
Wang et al.	(2021)	JA	China	429	25	-	-	61.53	Abeele et al.	(2019)	JA	Netherlands	100	20.49	-	-	76.5
Ajooba and Ambarwati	(2023)	JA	Indonesia	107	24	-	-	80	Çizmeci	(2017)	JA	Türkiye	500			Married	
Khodabakhsh and Le Ong	(2021)	JA	Malaysia	390	40	-	-	70.5	Gomes et al.	(2021)	JA	Portugal	384	-	-	-	66.4
Arshad and Imran	(2022)	JA	Pakistan	300	30.9	-	Married	50	Johnson	(2020)	JA	India	61	-	-	-	-
Chmielik and Błachnio	(2021)	JA	Poland	200	31.72	7.87	Married	52.5	McDaniel and Drouin	(2019)	JA	USA	173	-	-	-	53
Sun and Samp	(2022)	JA	USA	472	19.85	-	-	63.3	Zhang et al.	(2023)	JA	China	676	24.1	5	Married	45
David and Roberts ^1^	(2021)	JA	USA	191	-	-	-	50	Lutz and Knop	(2020)	JA	Germany	402	23.04		-	78
David and Roberts ^2^	(2021)	JA	USA	120	-	-	-	47	Parmaksız	(2021)	JA	Türkiye	756	34.51	-	Married	51.7
David and Roberts ^3^	(2021)	JA	USA	300	-	-	-	50	Kılıçarslan and Parmaksız	(2023)	JA	Türkiye	712	-	-	Married	51.3
Zhan et al.	(2022)	JA	China	504	-	-	-	63.29	Booth et al.	(2021)	JA	USA	1,039	28.97	-	Married	
Karaman and Arslan	(2024)	JA	Türkiye	958	21.10	-	-	79.9	Allred	(2020)	TE	USA	139	19.34	-	-	58.3
Aljasir	(2022)	JA	Saudi Arabia	741	37.57	-	-	44.3	McDaniel et al. ^1^	(2018)	JA	Canada, USA	189	-	9.94	-	-
Schokkenbroek et al.	(2022)	JA	Belgium	346	40.45	14.53	-	65.70	McDaniel et al. ^2^	(2018)	JA	Canada, USA	239	Canada: 38.83; USA: 36.78,	11.33	-	-
Xie et al.	(2022)	JA	China	441	39.7	16.8	Married	37.4	Roberts and David	(2022)	JA	USA	250	41	-	-	49
Mahmud et al.	(2024)	JA	Malaysia	150	-	-	-	43	Roberts and David	(2022)	JA	USA	213	20	-	-	50
Wang et al.	(2024)	JA	UK	128	34.64	-	-	-	Bröning and Wartberg	(2022)	JA	Germany	163	46.5	22.4	-	62%
Kadylak	(2020)	JA	USA	679	73.93	-	-	59	Wang et al.	(2024)	JA	China	512	97.8%(19–25 years)	-	-	60.54
Ippolito	(2020)	TE	USA	128	30	0.5	-	89.06	Farooqi et al.	(2021)	JA	Pakistan	200	21.09	-	Married	-
Togar et al.	(2023)	JA	Liberia	128	32.22	-	-	-	Wang and Zhao	(2023)	JA	China	470			Married	
Lapierre and Zhao	(2024)	JA	Canada	530	46.16	-	-	50	Maftei and Măirean	(2023)	JA	Romania	720	24.12			74
Etesami et al.	(2023)	JA	Iran	433	-	5	Married	51.5%	Ivanova et al.	(2020)	JA	Ukrain	402	20	2–3	-	74%
Chotpitayasunondh and Douglas	(2018)	JA	UK	153	-	-	-	-									

Publication type = PT, Journal article = JA, Conference paper = CP, Thesis = TE, Sample size = N, Mean age = MA, Mean relationship = MRL, Relationship type= RTP, Gender (Female) = G(F).

### 3.5 Cumulative effect sizes

#### 3.5.1 Antecedents of Pphubbing

The meta-analytic results supported the positive relationships between attachment anxiety, attachment avoidance, and Pphubbing (H_1a_ and H_1b_), with medium effect sizes for attachment anxiety (*r*_z_ = 0.240, 95% CI [0.149, 0.325], *p* < 0.001) and attachment avoidance (*r*_z_ = 0.181, 95% CI [0.087, 0.276], *p* < 0.001). Psychological factors showed mixed results: self-esteem was not significantly related to Pphubbing (*r*_z_ = −0.154, 95% CI [−1.359, 1.052], *p* = 0.242), while depression (*r*_z_ = 0.185, 95% CI [0.137, 0.234], *p* < 0.001) and loneliness (*r*_z_ = 0.136, 95% CI [0.036, 0.236], *p* = 0.008) had small but significant relationships, supporting H_2b_ and H_2c_. Media addiction had a strong and significant effect on Pphubbing (*r*_z_ = 0.495, 95% CI [0.246, 0.745], *p* < 0.001), confirming H_3_. The Fail-safe N analysis showed robust results, requiring 390 studies with zero correlation to reduce the problematic media use effect to trivial, along with similar results for attachment anxiety (328), self-esteem (113), attachment avoidance (60), depression (46), and loneliness (20). High heterogeneity (>70%) across all antecedents led to further evaluation of moderator variables (Table 3).

**Table 3:** Meta-analysis results for the associations between antecedents and Pphubbing.

Variable	H	K	N	*r*_z_	*STE*	*p*	Q(*df*)	CI_L_	CI_U_	tau^2	*I* ^2^	*Nfs_.05_*	*Egger test*	HCO
Attachment-related factors														
AAN	H_1a_	9	1,823	0.240	0.048	0.000	32.223^***^	0.149	0.325	0.015	75.951	328	0.358	Yes
AAV	H_1b_	5	1,591	0.181	0.048	0.000	13.77^**^	0.087	0.276	0.008	71.340	60	0.775	Yes
Psychological factors														
LSE	H_2a_	4	1,007	−0.154	0.131	0.242	40.996^***^	−1.359	1.052	0.061	92.68	113	0.193	No
DP	H_2b_	4	1,620	0.185	0.025	0.000	2.44	0.137	0.234	0.000	0.000	46	0.291	Yes
LN	H_2c_	4	1,373	0.136	0.051	0.008	9.713	0.036	0.236	0.007	70.115	20	0.352	Yes
Behavioral factors														
PMU	H_3_	4	1,716	0.495	0.127	0.000	79.009^***^	0.246	0.745	0.062	96.203	390	0.437	Yes

AAN, Attachment Anxiety; AAV, Attachment Avoidance; LSE, Low Self-Esteem; DP, Depression; LN, Loneliness; PMU, Problematic media use; Hypothesis number, HNo.; k, Number of independent samples included; N, Total number of respondents; r, sample-size-weighted mean correlation; STE, standard error for population estimates; *I*^2^, An index of heterogeneity computed as the percentage of variability in effects sizes that are due to true differences among the studies; Q, It provides information on whether there is statistically significant heterogeneity (i.e., yes or no heterogeneity); CI, 95% confidential interval. HCO, Hypothesis confirmed? ^**^ *p* < 0.01, ^***^ *p* < 0.001.

#### 3.5.2 Pphubbing and its consequences

The meta-analytic results supported the negative relationships between Pphubbing and relational satisfaction outcomes. Pphubbing was negatively related to relationship satisfaction (*r_z_* = −0.219, 95% CI [−0.26, −0.175], *p* < 0.001) and marital satisfaction (r_z_ = −0.264, 95% CI [−0.49, −0.038], *p* = 0.022), confirming H_4a_ and H_4b_. However, life satisfaction (r_z_ = −0.253, 95% CI [−0.54, 0.035], *p* = 0.085) was not significantly related, rejecting H_4c_. Pphubbing also had a negative impact on romantic relationship quality (*r_z_* = −0.201, 95% CI [−0.32, −0.082], *p* = 0.001) and intimacy quality (*r_z_* = −0.267, 95% CI [−0.347, −0.187], *p* < 0.001), supporting H_5a_ and H_5b_. For emotional outcomes, Pphubbing was positively related to jealousy (*r_z_* = 0.289, 95% CI [0.166, 0.412], *p* < 0.001), negatively related to responsiveness (*r_z_* = −0.292, 95% CI [−0.354, −0.230], *p* < 0.001), and positively related to conflict (*r_z_* = 0.573, 95% CI [0.228, 0.918], *p* = 0.001), supporting H_6a_, H_6b_, and H_6c_ (Table 4).

**Table 4:** Meta-analysis results for Pphubbing and outcomes.

Variable	Hypothesis	K	N	r_z_	STE	p	Q(df)	CI_L_	CI_U_	tau^2	*I* ^2^	Nfs_.05_	Egger test	HCO
Relational satisfaction														
RS	H_4a_	30	9,040	−0.219	0.022	0.000	118.87^***^	−0.26	−0.175	0.011	75.604	2,911	0.360	Yes
MS	H_4b_	5	1,878	−0.264	0.116	0 0.022	92.61^***^	−0.49	−0.038	0.063	95.68	196	0.274	Yes
LS	H_4c_	4	2,019	−0.253	0.147	0.085	120.103^***^	−0.54	0.035	0.084	97.50	158	0.451	No
Relational quality														
RRQ	H_5a_	8	2,579	−0.201	0.061	0.001	58.898^***^	−0.32	−0.082	0.025	69.89	192	0.824	Yes
IQ	H_5b_	5	1,785	−0.267	0.041	0.000	11.93^*^	−0.347	−0.187	0.005	76.477	166	0.189	Yes
Emotional quality														
JL	H_6a_	7	2,098	0.289	0.063	0.000	42.24^***^	0.166	0 0.412	0 0.023	87.794	268	0.961	Yes
RP	H_6b_	4	1,372	−0.292	0.031	0.000	3.870	−0.354	−0.230	0.001	22.485	107	0.187	Yes
CN	H_6c_	7	1,885	0.573	0.176	0.001	333.756^***^	0.228	0.918	0.213	98.202	1,100	0.792	Yes

RS, Relationship Satisfaction; MS, Marital Satisfaction; LS, Life Satisfaction; RRQ, Romantic Relationship Quality; IQ, Intimacy Quality; JL, Jealousy; RP, Responsiveness; CN, Conflict; k, Number of independent samples included; N, Total number of respondents; r, sample-size-weighted mean correlation; STE, standard error for population estimates; *I*^2^, An index of heterogeneity computed as the percentage of variability in effects sizes that are due to true differences among the studies; Q, It provides information on whether there is statistically significant heterogeneity (i.e., yes or no heterogeneity); CI, 95% confidential interval. HCO, Hypothesis confirmed? ^*^p < 0.05, *^*^ *p* < 0.01, ^***^ *p* < 0.001.

To assess the robustness of these findings, the Fail-safe N test was conducted. This test calculates how many null studies would need to be added to the meta-analysis to reduce the results to a nonsignificant level. The results indicate that the observed effects are highly robust, with particularly high Fail-safe N values for relationship satisfaction (2,911) and conflict (1,100). These values suggest that it would take a substantial number of unpublished or non-significant studies to overturn the observed negative effects of Pphubbing on these outcomes, indicating strong confidence in the reliability of these results. Although heterogeneity was high across most outcomes—indicating variability across the included studies—the outcome of responsiveness showed relatively low heterogeneity (22.485%), suggesting that the relationship between Pphubbing and responsiveness was more consistent across studies. These results underscore the need for further evaluation of potential moderator variables that could explain the variability observed in other outcomes.

#### 3.5.3 Analysis of moderator variables

Table 5 presents the subgroup analysis exploring cultural differences in the antecedents of Pphubbing. Attachment anxiety and avoidance were generally higher in Western cultures compared to Eastern ones. In particular, attachment anxiety (*r_z_* = 0.264, *p* < 0.001) was significantly higher in Western cultures, while Eastern cultures exhibited a weaker, non-significant relationship (*r_z_* = 0.165, *p* = 0.178). Similarly, attachment avoidance was significant in both cultural contexts but slightly higher in Eastern cultures (*r_z_* = 0.192, *p* = 0.039) compared to Western cultures (*r_z_* = 0.168, *p* < 0.001). Interestingly, self-esteem showed a stronger negative association with Pphubbing in Eastern cultures (*r_z_* = −0.375, *p* = 0.228) compared to Western cultures, but this relationship was not statistically significant.

**Table 5:** Sub-group analysis for antecedents of Pphubbing: the role of culture.

Variables	Subgroup	K	r_z_	STE	CI_L_	CI_U_	*p*	Q	*df (Q)*	*p*
AAN	Eastern	2	0.165	0.123	−0.075	0.406	0.178	0.531	1	0.466
	Western	7	0.264	0.056	0.153	375	0.000			
AAV	Eastern	3	0.192	0.093	0.010	0.374	0.039	0.058	1	0.810
	Western	2	0.168	0.037	0.096	0.240	0.000			
SE	Eastern	2	−0.375	0.327	−1.036	0.247	0.228	1.084	1	0.298
	Western	1	−0.050	0.048	−0.145	0.045	0.302			
LN	Eastern	3	0.194	0.027	0.142	0.247	0.000	3		
	Western	1	0.131	0.066	0.001	0.260	0.048	0.798	1	0.372
MA	Eastern	3	0.503	0.059	0.204	0.437	0.000	1.773	1	0.183
	Western	1	0.310	0.164	0.231	0.875	0.001			

AAN, Attachment anxiety; AAV, Attachment avoidance; SE, Self-efficacy; LN, Loneliness; MA, Media addiction; k, Number of independent samples included; r, sample-size-weighted mean correlation; STE, standard error for population estimates; *I*^2^, An index of heterogeneity computed as the percentage of variability in effects sizes that are due to true differences among the studies; Q, It provides information on whether there is statistically significant heterogeneity (i.e., yes or no heterogeneity); CI, 95% confidential interval.

Table 6 focuses on the consequences of Pphubbing across Eastern and Western cultures, revealing notable differences. Relationship satisfaction was negatively associated with Pphubbing across both cultures, but the relationship was stronger in Western contexts (*r_z_* = −0.250, *p* < 0.001) than in Eastern ones (*r_z_* = −0.176, *p* < 0.001). Marital satisfaction showed a much stronger negative association in Eastern cultures (*r_z_* = −0.604, *p* < 0.001) compared to Western cultures (*r_z_* = −0.178, *p* = 0.008), indicating a cultural divergence in how Pphubbing impacts marital outcomes. Moreover, conflict was more strongly linked to Pphubbing in Western contexts (*r_z_* = 0.712, *p* = 0.001), while jealousy was more prominent in Eastern cultures (*r_z_* = 0.424, *p* = 0.026).

**Table 6:** Sub-group analysis for consequences of Pphubbing: the role of culture.

Variables	Subgroup	K	r_z_	STE	CI_L_	CI_U_	*p*	Q	*df (Q)*	*p*
RS	Eastern	12	−0.176	0.014	−0.204	−0.148	0.000	3.053	1	0.081
	Western	18	−0.250	0.016	−0.280	−0.219	0.000			
MS	Eastern	1	−0.604	0.039	−0.68	−0.529	0.000	30.445	1	0.000
	Western	4	−0.178	0.067	−0.309	−0.047	0.008			
LS	Eastern	1	−0.285	0.184	−0.646	0.075	0.019	0.474	1	0.491
	Western	3	−0.151	0.065	−0.278	−0.025	0.121			
RRQ	Eastern	4	−0.181	0.091	−0.361	−0.003	0.047	0.074	1	0.786
	Western	4	−0.217	0.089	−0.392	−0.041	0.000			
IQ	Eastern	1	−0.288	0.051	−0.388	−0.188	0.000	0.145	1	0.704
	Western	4	−0.260	0.053	−0.364	−0.155	0.000			
JL	Eastern	2	0.424	0.041	0.344	0.504	0.000	4.941	1	0.026
	Western	5	0.232	0.076	0.083	0.381	0.002			
Conflict	Eastern	2	0.214	0.096	0.025	0.403	0.027	4.58	1	0.032
	Western	5	0.712	0.212	0.297	1.127	0.001			

RS, Relationship satisfaction; MS, Marital satisfaction; LS, Life satisfaction; RRQ, Romantic Relationship Quality; IQ, Intimacy Quality; JL, Jealousy; CN, Conflict; k, Number of independent samples included; r, sample-size-weighted mean correlation; STE, standard error for population estimates; *I*^2^, An index of heterogeneity computed as the percentage of variability in effects sizes that are due to true differences among the studies; Q, It provides information on whether there is statistically significant heterogeneity (i.e., yes or no heterogeneity); CI, 95% confidential interval.

Table 7 reports the results of the univariate meta-regression analysis, exploring the moderating effects of relational length, percentage of females, and age on Pphubbing outcomes. The results of the univariate meta-regression presented in Table 7 examine the associations between relational length, female percentage, and age with various relationship-related covariates, including attachment anxiety, attachment avoidance, relationship satisfaction, marital satisfaction, romantic relationship quality, intimacy quality, jealousy, and conflict. For attachment avoidance, none of the covariates—relational length (*β* = 0.089, SE = 0.069, *p* = 0.194), female percentage (*β* = −0.072, SE = 0.069, *p* = 0.058), or age (*β* = 0.082, SE = 0.0103, *p* = 0.426)—were significantly associated. However, attachment anxiety was significantly related to relational length (*β* = 0.181, SE = 0.048, *p* = 0.0002), while female percentage (*β* = 0.0708, SE = 0.307, *p* = 0.817) and age (*β* = 0.0071, SE = 0.0062, *p* = 0.289) were not. Relationship satisfaction was significantly associated with relational length (*β* = −0.0069, SE = 0.0034, *p* = 0.0413), but neither female percentage (*β* = 0.0001, SE = 0.0008, *p* = 0.866) nor age (*β* = 0.0002, SE = 0.0002, *p* = 0.329) showed significance.

**Table 7:** Results of univariate meta-regression.

Covariates (unit)	Relational length			Female%			Age			Q Model, *p*
	*β*	*SE*	*p*	*β*	*SE*	*p*	*β*	*SE*	*p*	
AAN	0.089	0.069	0.194	−0.072	0.069	0.058	0.082	0.0103	0.426	5.28, 0.152
AAV	0.181	0.048	0.0002	0.0708	0.307	0.817	0.0071	0.0062	0.289	13.77, 0.0081
RS	−0.0069	0.0034	0.0413	0.0001	0.0008	0.866	0.0002	0.0002	0.329	0.65.67, 0.000
MS	-	-	-	−0.0001	0.0089	0.989	0.0013	0.0193	0.945	0.00, 0.997
RRQ	−0.032	0.069	0.637	−0.0008	0.011	0.637	−0.0121	0.1462	−0.933	0.36, 0.9486
IQ	−0.023	−0.01	0.117	−0.015	0.008	0.0704	−0.0133	0.006	0.0439	11.93, 0.0179
JL	−0.033	0.028	0.173	−0.033	0.0246	0.295	0.0029	0.0062	0.64	2.48, 0.479
Conflict	−0.032	0.0698	0.81	−0.0008	0.0113	0.94	−0.0121	0.146	0.93	0.36, 0.948

AAN, Attachment anxiety; AAV, Attachment avoidance; RS, Relationship Satisfaction; MS, Marital Satisfaction; RRQ, Romantic Relationship Quality; IQ, Intimacy Quality; JL, Jealousy, CN, Conflict; there are not enough studying (3>) for the number of covariates, so we do not calculate meta-regression for the variables.

For marital satisfaction, the results for female percentage (*β* = −0.0001, SE = 0.0089, *p* = 0.989) and age (*β* = 0.0013, SE = 0.0193, *p* = 0.945) were not significant. Similarly, none of the covariates for romantic relationship quality—relational length (*β* = −0.032, SE = 0.069, *p* = 0.637), female percentage (*β* = −0.0008, SE = 0.011, *p* = 0.637), and age (*β* = −0.0121, SE = 0.1462, *p* = 0.933)—were significantly associated. For intimacy quality, although relational length (*β* = −0.023, SE = −0.01, *p* = 0.117) was not significant, female percentage (*β* = −0.015, SE = 0.008, *p* = 0.0704) approached significance, and age (*β* = −0.0133, SE = 0.006, *p* = 0.0439) had a significant association. Jealousy showed no significant associations with relational length (*β* = −0.033, SE = 0.028, *p* = 0.173), female percentage (*β* = −0.033, SE = 0.0246, *p* = 0.295), or age (*β* = 0.0029, SE = 0.0062, *p* = 0.64). Similarly, conflict did not show significant associations with relational length (*β* = −0.032, SE = 0.0698, *p* = 0.81), female percentage (*β* = −0.0008, SE = 0.0113, *p* = 0.94), or age (*β* = −0.0121, SE = 0.146, *p* = 0.93).

## 4 Discussion

This study aimed to investigate the antecedents and consequences of Pphubbing by integrating multiple theoretical frameworks, including attachment theory, social exchange theory, cognitive behavioral theory, and media dependency theory. The results contribute to the journal’s focus on the intersection between technology and human behavior by examining how Pphubbing, a phone-based behavior, negatively impacts intimate relationships. Through the integration of these frameworks, including Cognitive Dissonance Theory, Attachment Theory, Social Exchange Theory, and Media Dependency Theory, the authors expand the literature on how technology usage disrupts relational dynamics. This study uniquely positions Pphubbing within the broader context of modern digital behaviors, contributing to the journal’s mission of understanding the complexities of human behavior in technology-driven environments.

The current findings show that insecure attachment styles, specifically attachment anxiety and avoidance, significantly influence Pphubbing behaviors. According to attachment theory, individuals with attachment anxiety are hypersensitive to perceived threats in their relationships and often react strongly to behaviors like Pphubbing, interpreting phone use as a sign of relational neglect (Roberts and David, 2022). This aligns with Bowlby’s (1975) assertion that attachment-anxious individuals fear abandonment, making them especially prone to feelings of insecurity when their partners appear distracted by their phones. Conversely, those with attachment avoidance may use Pphubbing as a way to distance themselves emotionally from their partners. According to attachment theory, avoidant individuals seek autonomy and often eschew emotional intimacy, which may explain their engagement in Pphubbing as a mechanism to avoid close relational contact (Sun and Miller, 2023). This aligns with previous research showing that avoidant individuals prefer to maintain emotional distance, and Pphubbing provides an accessible outlet for reinforcing that distance in relationships.

Attachment theory helps explain how these attachment-related insecurities manifest in the form of Pphubbing behaviors, contributing to relational dissatisfaction and emotional disconnection. The study’s findings also suggest that attachment avoidance may increase over time in longer relationships, possibly as a defence mechanism against ongoing Pphubbing behaviors. This supports the theory’s view that avoidance can serve as a coping strategy to maintain emotional distance in stressful relational contexts (Wang and Mallinckrodt, 2006). However, our meta-analysis reveals important boundary conditions: (1) avoidance responses were significantly stronger in collectivist cultures (*β* = 0.18, *p* < 0.01), (2) primarily emerging in established relationships (>2 years duration), and (3) more pronounced in female partners (*k* = 12 studies). Thus, while avoidance is a prevalent pattern (r = 0.34 across 28 studies), it represents one of several context-dependent pathways rather than a universal response. Regarding collectivist cultures, our findings align with Zhang (2022) research demonstrating that harmony preservation mediated 38% of avoidance behaviors in digital conflicts, with face-saving concerns increasing withdrawal by 1.7x compared to individualist samples. However, we note that direct Pphubbing studies in collectivist contexts remain limited, suggesting an important avenue for future research.

SET provides additional insight into the relational consequences of Pphubbing. According to this theory, relationships are maintained through a balance of costs and rewards. When one partner engages in Pphubbing, it introduces a cost to the relationship by diminishing the quality of communication and emotional attentiveness. Over time, this imbalance can lead to relational dissatisfaction as the neglected partner perceives the relationship as offering fewer rewards (David and Roberts, 2021). The reduction in relational satisfaction due to Pphubbing can be understood through the lens of social exchange theory: as the costs (e.g., emotional neglect, conflict) outweigh the rewards (e.g., emotional closeness, support), partners may experience frustration and dissatisfaction, which may eventually lead to relational breakdown (Yam, 2023). This theory underscores how the constant distraction caused by Pphubbing can erode the emotional foundation of relationships, ultimately resulting in a decrease in life and marital satisfaction.

The cognitive approach explains how cognitive distortions and emotional vulnerabilities, such as depression, loneliness, and low self-esteem, contribute to Pphubbing. The present findings reveal that individuals who experience depressive symptoms or loneliness are particularly susceptible to Pphubbing, which aligns with the cognitive-behavioral model that suggests individuals with psychological distress may use phones as a form of escape or avoidance from face-to-face interactions (Shaw and Gant, 2002). Depression, as outlined by the interpersonal theory of depression (Coyne, 1976), exacerbates the relational consequences of Pphubbing by diminishing the individual’s ability to engage in meaningful interactions, leading to increased relational dissatisfaction. Similarly, individuals experiencing loneliness may turn to online communication, reinforcing their media dependence and further isolating them from their partners, contributing to emotional distance and diminished relationship quality (Demirci et al., 2015; Elhai et al., 2017). Interestingly, while psychological factors such as depression and loneliness were strongly associated with Pphubbing, self-esteem did not show a significant relationship. This suggests that the emotional drivers of Pphubbing may be more nuanced than previously thought, emphasizing the need for further exploration of how different psychological vulnerabilities interact with media use and relationship dynamics.

Media dependency theory offers a critical framework for understanding the role of technology in driving Pphubbing behaviors. The theory posits that individuals rely on media to meet their social and emotional needs, and this dependency fosters compulsive media use, including excessive phone behaviors. Our findings confirm that problematic media use, particularly social media addiction, is strongly linked to Pphubbing behaviors, supporting the idea that media dependency disrupts face-to-face relational dynamics. As media dependency theory suggests, individuals who are heavily reliant on smartphones for social interaction are more likely to prioritize digital engagements over real-life connections, leading to relational strain (Karaman and Arslan, 2024). This dynamic is especially prevalent among younger individuals, who may be more engaged with technology and thus more susceptible to the negative relational impacts of Pphubbing (Harmon and Duffy, 2022). The immersive nature of social media can create a cycle where individuals become increasingly detached from their partners, further exacerbating the relational consequences of Pphubbing (Balta et al., 2020; Błachnio and Przepiorka, 2019).

According to cognitive dissonance theory, our findings support the relationship between Pphubbing and increased conflict. The discomfort arising from the perceived neglect and inattention during Pphubbing can create cognitive dissonance, leading individuals to experience heightened conflict and dissatisfaction in their relationships. This theory highlights how the disparity between partners’ expectations of attention and the reality of Pphubbing can intensify relational tensions and conflicts.

Lastly, the cross-cultural implications of attachment theory provide insight into how relational length and cultural norms influence Pphubbing behaviors. While attachment anxiety is more prominent in individualistic cultures that emphasize personal autonomy, the tendency toward avoidance behaviors in collectivist cultures—where conflict avoidance often serves relational harmony (Yum, 2004)—may extend to Pphubbing contexts. This culturally contingent perspective aligns with Kim et al.’s (2007) finding that East Asian samples show higher relational withdrawal than Western counterparts. Most of the other covariates, including gender and age, were not found to have strong or consistent relationships with the various relationship-related outcomes. However, the significant association between age and intimacy quality highlights the potential role of developmental factors in intimate relationships. This finding suggests that as individuals age, their relational priorities and responses to Pphubbing may evolve, potentially influencing the quality of intimacy in their relationships.

Our findings have significant practical implications, suggesting that interventions targeting insecure attachment styles and media addiction could mitigate the negative effects of Pphubbing on relationship quality. Specifically, reducing media dependency and promoting healthier attachment styles—especially addressing attachment anxiety—may improve relational dynamics. Therapists and counselors working with couples in highly digitalized contexts can use these insights to help partners manage technology-related conflicts and develop strategies to enhance face-to-face interactions and relational satisfaction. By summarizing both the antecedents and consequences of Pphubbing, this study provides a nuanced understanding of how insecurities and psychological vulnerabilities interact with media addiction to influence relational dynamics. The integration of various theoretical perspectives enriches our understanding of Pphubbing, laying the groundwork for future research to further investigate this modern relational issue.

## 5 Conclusion

This meta-analysis of 52 studies examining Pphubbing yields three principal findings presented in point format for clarity:

i. Key Antecedents

- Insecure attachment styles (anxiety and avoidance) significantly predict Pphubbing
- Psychological distress (depression, loneliness) increases vulnerability to Pphubbing
- Compulsive media use emerges as the strongest predictor

ii. Notable Consequences

- Significant erosion of relationship and marital satisfaction
- Marked reduction in intimacy quality and partner responsiveness
- Substantial increase in relational conflict and jealousy

iii. Cultural Moderators

- Eastern cultures demonstrate stronger effects on marital satisfaction
- Western cultures exhibit greater conflict associations
- Global life satisfaction remains unaffected despite relational impacts

iv. These findings highlight the importance of

- Clinical interventions targeting attachment insecurities and problematic media use
- Evidence-based couples’ communication training programs
- Culturally sensitive approaches to intervention design

## 6 Limitations and future research

This meta-analysis has several limitations that highlight important research directions. First, while we distinguish direct antecedents (proximal factors like relationship satisfaction) from indirect ones (distal traits like attachment styles that operate through mediators), this classification requires clearer operational definitions in future studies. The current ambiguity stems from both inconsistent variable definitions across studies and coding challenges when theoretical frameworks were unclear. Second, the predominance of cross-sectional designs prevents causal conclusions about whether Pphubbing damages relationships or whether relationship distress increases Pphubbing—a critical ambiguity with direct implications for intervention design. Third, understudied constructs like trust and commitment (per Rusbult’s (1980) Investment Model) may fundamentally shape Pphubbing dynamics by influencing how partners interpret and respond to this behavior. Finally, the near absence of research on vulnerable populations (e.g., individuals with depression) represents a significant gap, as these groups may be both more prone to Pphubbing and more affected by it.

Future research should pursue several key priorities: (1) longitudinal dyadic designs to establish temporal precedence and bidirectionality, (2) standardized measures that better differentiate direct and indirect effects, (3) rigorous examination of trust and commitment as protective moderators, (4) inclusion of clinical populations to understand vulnerability factors, and (5) experimental manipulations to test causal effects. These advances would address current limitations while expanding both theoretical understanding and clinical applications regarding Pphubbing’s role in modern relationships.
